# FARCI: Fast and Robust Connectome Inference

**DOI:** 10.3390/brainsci11121556

**Published:** 2021-11-24

**Authors:** Saber Meamardoost, Mahasweta Bhattacharya, Eun Jung Hwang, Takaki Komiyama, Claudia Mewes, Linbing Wang, Ying Zhang, Rudiyanto Gunawan

**Affiliations:** 1 Department of Chemical and Biological Engineering, University at Buffalo, Buffalo, NY 14260, USA; sabermea@buffalo.edu; 2 Department of Biomedical Engineering, University at Buffalo, Buffalo, NY 14260, USA; mahaswet@buffalo.edu; 3 Neurobiology Section, Center for Neural Circuits and Behavior, Department of Neurosciences, University of California San Diego, La Jolla, CA 92093, USA; eunjunghwang.phd@gmail.com (E.J.H.); tkomiyama@ucsd.edu (T.K.); 4 Cell Biology and Anatomy Discipline, Center for Brain Function and Repair, Chicago Medical School, Rosalind Franklin University of Medicine and Science, North Chicago, IL 60064, USA; 5 Department of Physics and Astronomy, University of Alabama, Tuscaloosa, AL 35487, USA; ckmewes@ua.edu; 6 Department of Civil and Environmental Engineering, Virginia Polytechnic Institute and State University, Blacksburg, VA 24061, USA; wangl@vt.edu; 7 Department of Cell and Molecular Biology, University of Rhode Island, Kingston, RI 02881, USA; yingzhang@uri.edu

**Keywords:** connectome inference, functional connectome, two-photon Ca^2+^ imaging, Neural Connectomics Challenge

## Abstract

The inference of neuronal connectome from large-scale neuronal activity recordings, such as two-photon Calcium imaging, represents an active area of research in computational neuroscience. In this work, we developed FARCI (Fast and Robust Connectome Inference), a MATLAB package for neuronal connectome inference from high-dimensional two-photon Calcium fluorescence data. We employed partial correlations as a measure of the functional association strength between pairs of neurons to reconstruct a neuronal connectome. We demonstrated using in silico datasets from the Neural Connectomics Challenge (NCC) and those generated using the state-of-the-art simulator of Neural Anatomy and Optimal Microscopy (NAOMi) that FARCI provides an accurate connectome and its performance is robust to network sizes, missing neurons, and noise levels. Moreover, FARCI is computationally efficient and highly scalable to large networks. In comparison with the best performing connectome inference algorithm in the NCC, Generalized Transfer Entropy (GTE), and Fluorescence Single Neuron and Network Analysis Package (FluoroSNNAP), FARCI produces more accurate networks over different network sizes, while providing significantly better computational speed and scaling.

## 1. Introduction

The human brain comprises about 100 billion neurons that are communicating with one another via more than a quadrillion synaptic connections. The brain’s functional connectome, the connectivity of neurons or brain areas as a functioning network, is highly plastic and dynamic, a feature that imparts the brain with an ability to learn new behavior and to store and process new information [1]. The reconstruction of the brain’s functional connectivity has received much attention for elucidating the operating principles of the brain and its myriad functions, as well as their dysfunctions in neurological diseases.

Direct identification of neuronal connectivity based on anatomical measurements are time-consuming, non-scalable, and challenging due to various limitations of macroscale imaging modalities [2,3,4]. For this reason, functional connectomes are typically reconstructed from neuronal activity recording data. Technological advances in neuroscience have enabled the recording of neuronal activity signals in awake animals [5], accelerating efforts toward functional connectome inference [6]. Of note, two-photon (2p) Calcium imaging provides in vivo optical measurements of neuronal firing [7], with recent technology capable of simultaneous recording of ~10,000 neurons [8,9]. The technique relies on fluorometric Ca^2+^ indicators, either using chemical dyes or genetically encoded Ca^2+^ indicator (GECI), to detect Ca^2+^ transients in a neuron associated with an action potential. When combined with powerful image processing algorithms, 2p Ca^2+^ imaging enables long-term monitoring of the activity of neuronal ensembles in awake animals, and how the activity and thus functional connectome of these neurons change over time, for example, with learning [5,7,10,11]. Note that further data processing is needed to infer neuronal action potentials from Ca^2+^ fluorescence traces. Extracting neuronal activity from Ca^2+^ imaging data is a non-trivial task due to significant noise, baseline fluorescence drift, and other technical constraints, such as low sampling rate and slow decay of fluorescence sensor relative to the time-scale of neuronal firing dynamics [12]. Finally, we still lack technologies that are able to record the activity of all neurons in the brain simultaneously in complex organisms such as rodents or primates (i.e., some neurons are hidden from the measurements). Thus, functional connectome inferred from neuronal activity data does not necessarily imply the existence of (actual) synaptic connections between neurons.

State-of-the-art computational algorithms have been developed to reconstruct the functional connectome from Calcium imaging data. The existing methods can be classified into two classes: model-free methods and model-based methods (see [6] for a more comprehensive review). In general, model-free methods are computationally simpler than model-based strategies, and thus, are more amenable for large-scale connectome inference. Model-free methods use statistical associations of neuronal firing activity to establish functional connectivity among neurons. Descriptive statistics such as Pearson correlations and partial correlations are commonly used in model-free methods because of their simplicity [13]. However, such metrics are only able to describe linear and non-causal (undirected) associations among neurons. More sophisticated methods use information theoretic measures such as mutual information to capture non-linear functional associations. Further, by considering information flow using transfer entropy, such methods are also able to extract directional connections [14,15]. Finally, following recent successes of deep learning in various applications, supervised learning strategies such as convolutional neural networks have been applied to infer functional connectome directly from two-photon Calcium fluorescence images [16]. However, more advanced methods that use information theory and deep learning have higher computational and data requirements than those using descriptive statistics. Also, despite their simplicity, correlative metrics were among the top performing methods in the Neural Connectomics Challenge (NCC) [17].

In this work, we developed a model-free method FARCI (Fast and Robust Connectome Inference), a MATLAB toolbox for inferring functional connectome from time-series Calcium fluorescence data of the neuronal activity. In developing FARCI, we combined non-negative spike deconvolution, thresholding, and smoothing for data pre-processing and employed a partial correlation network of neuronal activity for functional connectome representation. The functional connectome inference pipeline was optimized to achieve good performance in functional connectome inference, computational efficiency and scalability, and robustness to missing neurons. We assessed the performance of FARCI using in silico Calcium fluorescence datasets from the Neural Connectomics Challenge (NCC) [17] and datasets generated using the state-of-the-art simulator of Neural Anatomy and Optical Microscopy (NAOMi) [18]. The performance of FARCI is compared with the winning method of NCC (Sutera et al.) [13] Generalized Transfer Entropy algorithm [15]—the baseline method in the NCC;and Fluorescence Single Neuron and Network Analysis Package (FluoroSNNAP) [19]—a Calcium image analysis toolbox that includes connectome inference. The results demonstrate that FARCI is highly efficient and scalable to large datasets and connectomes, and its performance is superior to the above comparative methods in terms of accuracy and robustness to noise levels, sampling rates, network densities, and hidden (missing) neurons.

## 2. Materials and Methods

### 2.1. Spike Deconvolution

Precise temporal information of individual neurons’ spiking activity is crucial for functional connectome inference. The inference of neuronal Ca^2+^ spikes from 2p fluorescence images is an active area of research with more sophisticated methods being developed in a regular fashion. In our work, we used sparse Non-Negative Deconvolution (NND) method for spike inference because of its relatively fast and robust performance, as demonstrated in a recent Spike Inference Challenge [12]. Other spike inference algorithms, such as MLSpike [20] or CASCADE [21], can also be used in place of the NND method, if desired.

FARCI uses a sparse NND from the Online Active Set method to Infer Spikes (OASIS) algorithm [22] within the Suite2P MATLAB package [23]. We write the neuronal spike deconvolution as follows:

(1) $$x_{i}=f\left(C_{i}\right)$$

where$\;C_{i}$ is the raw Ca^2+^ fluorescence data (dF/F0) of neuron *i*, f represents the deconvolution function, and $x_{i}$ is the deconvolved neuronal spiking activity.

### 2.2. Spike Thresholding

The deconvolved spiking activities are contaminated by noise and such noise can degrade the accuracy of the inferred connectome. For this reason, we remove any deconvolved neuronal spike activity $x_{i}$ of the *i*-th neuron that is below a certain threshold $\Theta_{i}$. The threshold is set to the average of $x_{i}$ plus a user-specified constant multiple α of the standard deviation of $x_{i}$. More specifically, the thresholded spiking activity, denoted by $y_{i}$, is computed as follows:

(2) $$y_{i}=g\left(x_{i}\right)$$

(3) $$g\left(x_{i}\right)=\{\begin{array}{c} 0,\;\;x_{i}<\Theta_{i}\\ x_{i},\;\;x_{i}\;\geq \Theta_{i} \end{array}$$

(4) $$\Theta_{i}=\mu_{i}+\alpha \times \sigma_{i}\;(\mathrm{default}\;\alpha =2)$$

where $\Theta_{i}$ is the neuron-specific threshold, and $\mu_{i}$ and $\sigma_{i}$ are the sample mean and standard deviation of $x_{i}$, respectively. While thresholding may remove true spiking activity with a low amplitude, our tests (see Results) indicate that the accuracy gain by spike thresholding outweighs the loss of information due to removal of low-amplitude spiking activity.

### 2.3. Spike Smoothing

**Figure 1:**
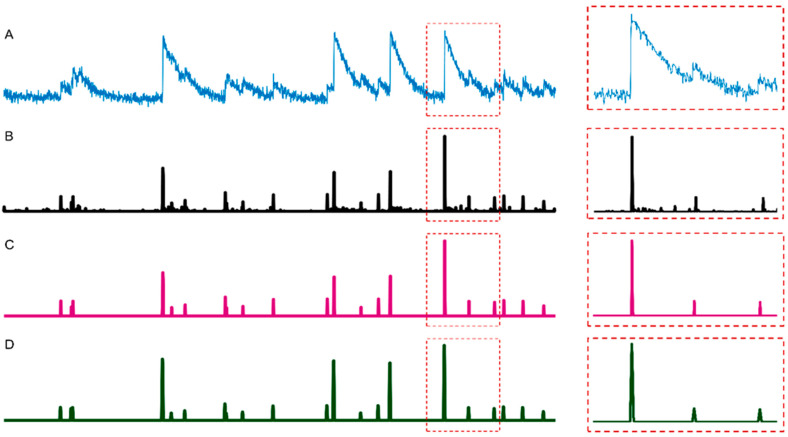
Data preprocessing pipeline of Calcium fluorescence data. (**A**) Raw Ca^2+^ fluorescence signal (dF/F0). (**B**) Deconvolved spikes using OASIS. (**C**) Thresholded spikes. (**D**) Smoothed spikes.

Smoothing spikes over multiple time points (image frames) have been shown to enhance the correlations between deconvolved and ground-truth spiking activity [24]. We tested different weighted smoothing strategies and identified the following smoothing function $h\left(y_{i},t\right)$ to give the best performance:

(5) $$z_{i}\left(t\right)=h\left(y_{i},t\right)$$

(6) $$h\left(y_{i},t\right)=\frac{1}{3}y_{i}^{t-2}+\frac{2}{3}y_{i}^{t-1}+y_{i}^{t}+\frac{2}{3}y_{i}^{t+1}+\frac{1}{3}y_{i}^{t+2}$$

where $z_{i}$ denotes the smoothed spikes and *t* denotes the time index. The weighting coefficients were assigned to give higher importance to data in the specific time *t*, while the weights for the neighboring two time points in both positive and negative directions were set in decreasing order according to the time distance. Note that the partial correlation calculation is not affected by the fact that the weights do not sum to one. The data pre-processing pipeline from Ca^2+^ fluorescence data to the final neuronal activity spikes is illustrated in Figure 1.

### 2.4. Partial Correlation Statistics

In FARCI, the functional connectivity between each pair of neurons is established based on their co-firing behavior. However, due to the highly interconnected nature of neurons, functional correlations between any two neurons may arise indirectly from their connections to other neurons (e.g., sharing the same pre-synaptic neurons). To reduce false positives, FARCI uses partial correlation coefficient as a measure of functional connectivity between a pair of neurons—that is, the correlation between the spike activity of two neurons while controlling for the activity of other neurons [25]. In order to infer an edge (connection) between two neurons, we evaluate the partial correlation $p_{ij}$ between neurons i and j via the precision matrix Φ, as follows:

(7) $$p_{ij}=-\frac{\Phi_{ij}}{\sqrt{\Phi_{ii}\Phi_{jj}}}$$

(8) $$\Phi =\Sigma^{-1}$$

where Σ is the N×N covariance matrix of neuronal activity among *N* neurons evaluated using the smoothed spikes $z_{i}$, and Φ is the precision matrix. The partial correlation coefficients have values between −1 and +1, where a value of +1 (−1) indicates a perfect positive (negative) correlation of spike activity between two neurons while controlling for the activity of other neurons.

### 2.5. Performance Evaluation 

We evaluated the performance of FARCI using in silico Ca^2+^ fluorescence datasets (dF/F0) from the Neural Connectomics Challenge and datasets generated using NAOMi simulator [18]. The NCC datasets were simulated using a mathematical model of Ca^2+^ fluorescence signal that takes into account limitations of Ca^2+^ imaging technology such as temporal resolution and light scattering artifacts [15,26]. In the NCC, the challenge organizers generated in silico Calcium fluorescence images for neurons that are placed randomly in a 1 mm × 1 mm area with random connections of a given average connectivity and clustering coefficient, which isthe average number of triangles a neuron forms with its neighbors over the total possible number of triangles it could form given its connectivity. A high clustering coefficient is associated with a network with tightly connected neighborhoods [18]. A model of leaky integrate and fire neuron with short term synaptic depression [26] was implemented in the NEST simulator [27,28] to generate neuronal firing dynamics with a firing rate of 0.1 Hz. The neuronal firing was then coupled with a fluorescence response model of Ca^2+^ markers to simulate in silico Ca^2+^ images at a rate of 50 Hz for 60 min. Ten baseline datasets for neuronal networks of size 100 (*n =* 6) and 1000 neurons (*n* = 4)—referred to as the small and normal connectomes, respectively—were generated (see Table 1). The connectivity of the small and normal connectomes has a relatively low density that ranges within 16.3±1.7% for the small networks and 2.1±0.5% for the normal networks, suggesting that these connectomes are sparse. Six additional datasets of 1000 neurons for higher and lower levels of signal-to-noise ratio, neuronal firing rate, network clustering coefficient, and average connectivity, were also available. More details of the data generation can be found in the NCC publication [17]. We also tested the performance of FARCI on a lower imaging rate of 25 Hz by downsampling the NCC datasets—keeping every other frame of the original data. 

Ten additional in silico datasets were generated using the NAOMi toolbox [18]. NAOMi enables the simulations of biologically realistic neural volume that includes vasculatures and neurons with soma, axons, and dendrites, and the corresponding time-series Ca^2+^ fluorescence images for the neuronal population in this volume. The number of neurons in the volume is generated randomly, and so is the neuronal connectome, specifically using the Hawkes model [29] based on the Watts–Strogatz small-world network [30]. Neuronal activity is modeled as correlated bursting neurons, which is coupled with an optical microscopy model to produce in silico Ca^2+^ fluorescence images. We utilized NAOMi to generate five neuronal volumes of size 50 μm × 50 μm × 150 μm with ~100 neurons and another five volumes of size 300 μm × 300 μm × 150 μm with ~1000 neurons. NAOMi simulation parameters that were used for in silico data generation are detailed in Supplementary Table S1. The generated in silico Ca^2+^ fluorescence images were converted to time-series fluorescence traces (dF/F0) using a built-in subroutine in NAOMi (times_from_prof).

For scoring the performance of FARCI and the comparative methods (see Section 2.6. Performance Comparison), we followed the strategy used in the NCC. Submissions from the participants were ranked based on how accurately their algorithms were able to infer the neural connectomes with 1000 neurons. Specifically, the scoring in the NCC was done by evaluating the area under the Receiver Operating Characteristic (AUROC)—the curve of true positive rate vs. false positive rate—by comparing the ranked list of predicted connectivity with the ground truth network [31]. In our case, the ranked list of predicted connectivity is the list of connections ordered in decreasing magnitude of partial correlation coefficients. The ROC is created by picking the top *k* predicted connections and computing the number of true positive (TP), false positive (FP), false negative (FN), and true negative (TN). The ROC curve is the plot of true positive rate (TPR, the ratio of the number of true positives to the number of connections in the ground truth) versus false positive rate (the ratio of the number of false positives to the number of connections in the ground truth) for increasing *k* (*k* = 1, 2, 3, …) in the ranked prediction list. Here, the number of TPs is the number of connections among the top *k* predicted connections that also exist in the ground truth connectome. Connections in the top *k* predictions that are not in the ground truth connectome correspond to FPs. Meanwhile, the number of connections in the ground truth connectome that are not in the top *k* predictions gives the number of TNs. Finally, the connections that are not in the ground truth connectome nor in the top *k* predictions are the TNs.

We computed the AUROCs using the perfcurve function in MATLAB [32]. Besides AUROC, we computed the area under the Precision-Recall (AUPR) curve as an additional performance metric, also using MATLAB perfcurve function. Precision is the ratio of the number of TPs to the sum of the numbers of TPs and FPs, while recall is equivalent to the TPR. AUROC and AUPR values range between 0 and 1, where a value of 1 indicates perfect prediction. Also, note that an AUROC of 0.5 is the expected performance for a random prediction. For sparse ground truth networks where the number of true connections is low in comparison to the number of all possible connections, AUPR is a more sensitive measure for the performance of network inference methods than AUROC [33,34], since AUPR takes into account the ratio between positives and negatives (i.e., class imbalance). For sparse networks, AUROC values generally tend to be very high (near 1). 

### 2.6. Performance Comparison

We compared FARCI with three connectome inference methods developed for Ca^2+^ fluorescence data: the best performing method in the NCC by Sutera et al. 2015 [13], the baseline method in the NCC called Generalized Transfer Entropy [15], and a widely-used Calcium fluorescence analysis toolbox called FluoroSNNAP [19]. Sutera et al. algorithm comprises a four-step signal processing pipeline (low-pass filter, high-pass filter, hard thresholding, and weighting), and similar to FARCI, also produces partial correlation networks. We implemented Sutera et al. algorithm in MATLAB with the aid of the original developer [13], and were able to reproduce the results of the algorithm independently. Like FARCI, we used partial correlation coefficients generated by Sutera et al. algorithm to give the ranked list of neuronal connections (descending order) for performance scoring.

The Generalized Transfer Entropy method applies an information theoretic concept called Transfer Entropy (TE) to connectome inference [15]. TE provides a measure of information flow between two time-series random processes. In establishing connectivity among neurons, the GTE method evaluates the TE between every pair of neurons in the population using their time-series traces (dF/F0). Here, we used a numerically efficient MATLAB implementation provided by the original developer of GTE (private communication). For performance evaluation, we used the calculated TE for all pairs of neurons to rank the neuronal connections (in descending order). FluoroSNNAP [19] is an open-source MATLAB toolbox for interactive and automated analysis of Ca^2+^ fluorescence images. FluoroSNAPP relies on the temporal synchrony of spiking events to establish connectivity between neurons. Here, we used the MATLAB subroutine PhaseSpike in FluoroSNNAP package to evaluate the phase difference $\Psi_{X,Y}\left(t\right)$ of pairs of neurons *X* and *Y*, using the thresholded spike times of the neurons (i.e., the output of spike thresholding step in FARCI) as inputs. We then applied the subroutine FC_phase in FluoroSNNAP to perform 100 repeated runs of Kolmogorov–Smirnov (KS) tests comparing the phase difference $\Psi_{X,Y}\left(t\right)$ against a random sample $\hat{\Psi_{X,Y}}\left(t\right)$ taken from a null distribution. In FluoroSNNAP, the functional connection between neurons *X* and *Y* is determined based on the 95th percentile of the *p*-values from the KS tests above. Correspondingly, for performance scoring, we used the 95th percentile of the *p*-values to rank neuronal connections (in ascending order).

## 3. Results

**Figure 2:**
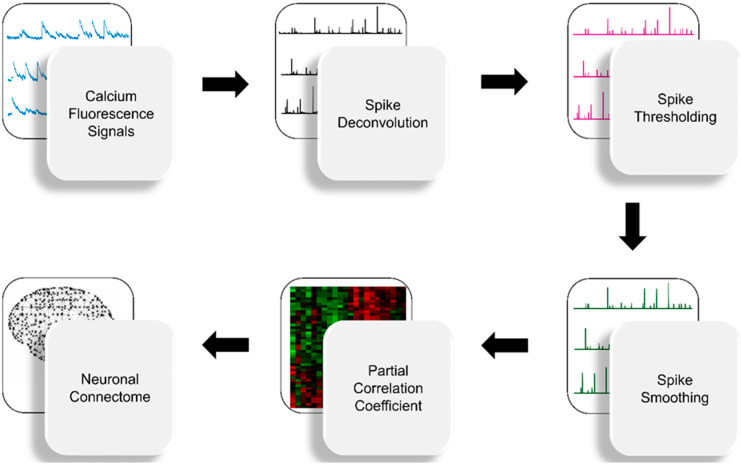
Workflow of connectome inference in FARCI. FARCI combines thresholding and smoothing of neuronal spikes, the output of which is used to generate partial correlation networks.

In this work, we developed FARCI, an efficacious and robust method for inferring functional neuronal connectome from Ca^2+^ fluorescence data. In FARCI, the functional neuronal connectome is represented by the partial correlation network among the neurons. Figure 2 illustrates the workflow of the functional connectome inference in FARCI, which comprises the following key steps: (1) deconvolution of spiking activity from Ca^2+^ fluorescence data, (2) spike thresholding, (3) spike smoothing, and (4) evaluation of partial correlations. The details of the individual steps can be found in Materials and Methods. We benchmarked FARCI using the Ca^2+^ fluorescence datasets from the Neural Connectomics Challenge (NCC) [17]. We also compared the performance of FARCI with that of the best performing method in the NCC, the inference algorithm by Sutera et al. [13].

### 3.1. Neuronal Spike Deconvolution

Ca^2+^ fluorescence imaging data give only indirect measurements of neuronal activity, and thus, require data pre-processing to extract the underlying neuronal action potential spikes. We employed the OASIS deconvolution algorithm [22] from the MATLAB package Suite2P [23] that uses a non-negative deconvolution strategy to provide estimates for timing and amplitude of spiking activity. Table 2 gives the AUROC and AUPR values for using the partial correlations of the deconvolved spikes to infer neuronal connectomes (see Supplementary Tables S2–S4 for more detailed results). While the AUROCs were generally good (>0.78), the AUPRs were as low as 0.23.

### 3.2. Binarization of Neuronal Spikes

We also tested whether binarizing the deconvolved spiking activity by setting non-zero spikes to 1 might help in improving the functional connectome inference using partial correlations. As reported in Table 2, converting spikes to binary data led to a significant deterioration in the accuracy of the inferred connectomes for both small and normal-sized networks. The result above suggests that the amplitude of spiking activity contains significant information for inferring neuronal connectivity. Thus, in FARCI, we used the deconvolved spiking activity without any binarization.

### 3.3. Neuronal Spike Thresholding

**Figure 3:**
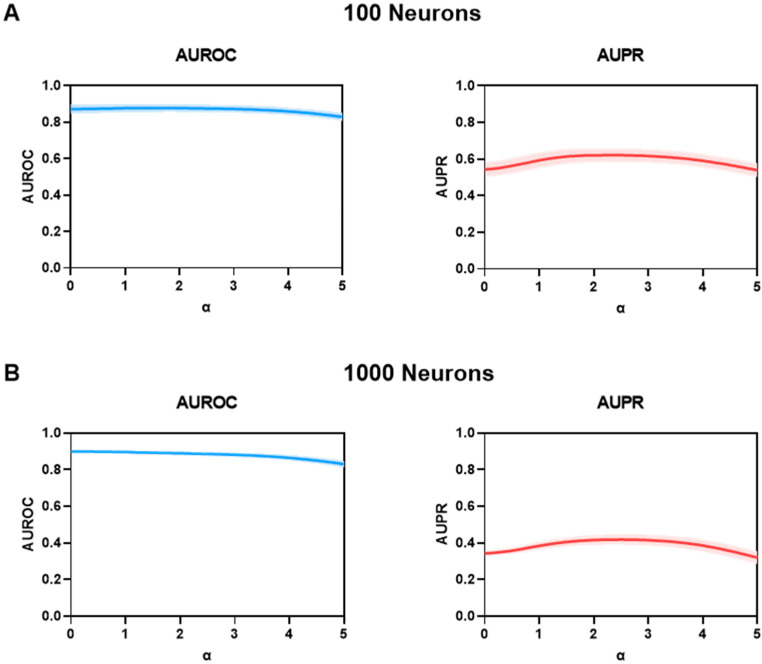
Effect of signal thresholding on AUROC and AUPR in networks of (**A**) 100 (*n* = 6) and (**B**) 1000 neurons (*n* = 10). While AUROC tends to drop with increasing α, AUPR reaches a peak for values of 2 < α < 3. The shaded area denotes the standard error of the mean (SEM).

Low amplitude spiking activity may arise from random noise and should ideally be filtered out to improve accuracy. In FARCI, we implemented a thresholding step by equating deconvolved spike activity heights that are lower than a neuron-specific threshold to 0 (see Section 2. Materials and Methods). The threshold was set to a user-defined multiple α of standard deviation above the average spike height for each neuron. In the following, we investigated the influence of the user-defined α on the AUROC and AUPR. Specifically, we ran the connectome inference using thresholded spikes for different α values in the range of 0≤α≤5. As shown in Figure 3, the AUROC generally drops with increasing thresholding strength (i.e., increasing α), especially for larger connectomes, but stays reasonably high at above 0.7. For large and sparse networks where the number of negative cases (i.e., the absence of neuronal connections) significantly outweighs the number of positive cases, AUROC often becomes too optimistic. Here, the AUPR serves as a more sensitive metric for method performance. The AUPRs for all of the connectomes show a peak for α between 2 and 3 with α=2 often giving the highest value. For this reason, we set α=2 as the default value for the spike thresholding step in FARCI. As reported in Table 2, the thresholding step using α=2 improves the AUPR on average by 18.6% over using only the deconvolved spike data directly. We also tested spike thresholding using a percentile cut-off (90th and 95th percentiles) with and without binarization (see Supplementary Figure S1). Again, binarization of spikes led to a poorer performance. Here, the 90th percentile cut-off gave a similar performance as using α=2 in the above.

### 3.4. Neuronal Spike Smoothing

Smoothing deconvolved spiking activity has been demonstrated previously to improve the connectome inference [13]. Similarly, binning spikes from OASIS increase the correlation between the predicted and ground truth spikes [24]. We explored a set of heuristic binning and smoothing functions for improving functional connectome inference accuracy (see Supplementary Tables S5 and S6), and identified the weighted binning given in Equation (6) as a simple-yet-efficacious smoothing function. The smoothing function uses a time window of five frames where higher weights are given to the time points closer to the center frame of the window (see Section 2. Materials and Methods). The performance of the connectome inference using smoothened spiking activity is reported in Table 2, which shows moderate improvements in the AUROC over that by using the spiking activity directly without binning. The weighting in the smoothing function in Equation (6) may have to be adjusted based on the imaging rate. As the imaging rate decreases, the weights should be more center-heavy—assigning more weights toward the center frame. The application to downsampled NCC datasets with a lower imaging rate of 25 Hz shows that the connectome inference performance remains relatively high (see Supplementary Figure S2).

### 3.5. FARCI Performance

**Figure 4:**
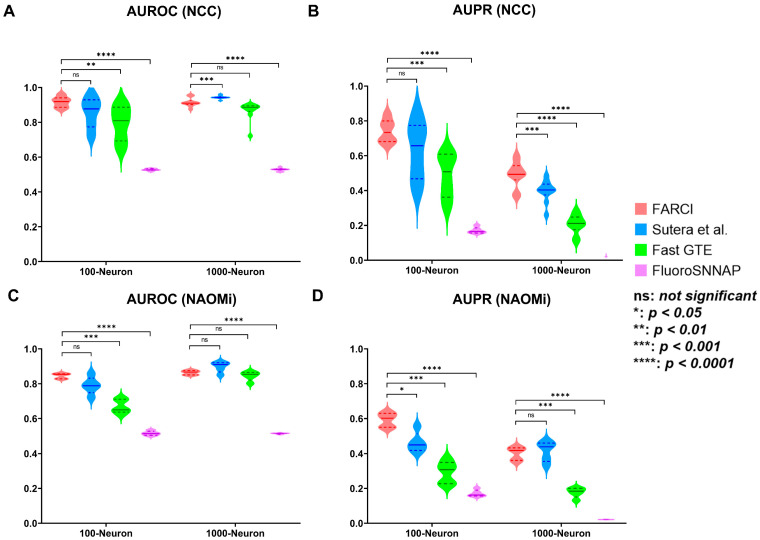
Performance comparison of FARCI with Sutera et al. GTE, and FluoroSNNAP algorithms. The accuracy of the inferred connectome is measured by (**A**,**C**) AUROC and (**B**,**D**) AUPR for (**A**,**B**) NCC and (**C**,**D**) NAOMi datasets. The complete results of the benchmarking and comparison are provided in Supplementary Tables S7 and S8. Statistical significance was assessed using two-sided paired t-test. The results for KS and F test for normality and constant variance are given in Supplementary Table S9.

FARCI combines thresholding and smoothing of the deconvolved spiking activity to produce a synergistic improvement in the connectome inference, as shown in Table 2. Importantly, the performance comparison in Figure 4 shows FARCI outperforming the best performing algorithm in the NCC by Sutera et al. [13], GTE [15], and FluoroSNNAP [19].

For the NCC datasets, FARCI is able to provide high AUROC and AUPR regardless of the size of the networks, level of noise, density of the networks, and neuronal firing frequency (see Figure 4A,B and Supplementary Table S7). Generally, FARCI and Sutera et al. had comparable performance, which is expected since both methods are based on partial correlations to establish neuronal connections. The algorithm by Sutera et al. produces higher AUROCs but lower AUPRs than FARCI for the normal connectomes with 1000 neurons, which was the network size used in the NCC method ranking. We noted that Sutera et al.’s algorithm performed poorer on the smaller connectomes with 100 neurons, suggesting a potential issue of overly optimized hyper-parameters. In addition, connectome inference based on partial correlations outperformed methods using transfer entropy (GTE) and spike phase (FluoroSNNAP). Notably, FluoroSNNAP gave the worst AUROCs and AUPRs with scores that resemble a random predictor (AUROC of 0.5).

We further tested the performance of FARCI and the comparative methods on in silico datasets generated using the state-of-the-art simulator NAOMi [18]. The results, as shown in Figure 4C,D are consistent with the outcomes of the applications of these methods to the NCC datasets in Figure 4A,B. Like before, FARCI and Sutera et al. provided comparably high AUROCs and AUPRs. Again, FARCI had a slight advantage over Sutera et al. algorithm in terms of AUPRs. In addition, GTE and FluoroSNNAP had worse performance than FARCI and Sutera et al. with FluoroSNNAP giving the lowest AUROCs and AUPRs among the methods (see detail results in Supplementary Table S8).

### 3.6. Missing Neurons

Finally, we investigated the robustness of FARCI with respect to missing or hidden neurons. The missing neurons can be considered as hidden variables in the connectome inference. Hidden variables are a common problem in functional connectome inference as only a subset of neurons can be measured in a typical experimental setup. Hidden variables may lead to false positives where a connection between two neurons is inferred when there is none in the ground truth. For example, when two neurons have one and the same presynaptic neuron, their activity would be perfectly correlated. But, the partial correlation between the two neurons, when controlling for the activity of the presynapse, is zero. Unfortunately, when the presynapse is missing from the ensemble (i.e., when it is not part of the neurons in the Ca^2+^ imaging plane), the two neurons may have a high partial correlation due to the lack of data for the presynapse.

In this work, we emulated missing neurons by randomly sampling a subset of neurons from the dataset, and then applied FARCI and the comparative methods to obtain the functional connectome for the subsampled dataset. We compared the inferred connectome with the subnetwork of the ground truth connectome corresponding to the randomly subsampled neurons. Here, we generated five random samples of neurons and their Ca^2+^ fluorescence data from the connectomes with 1000 neurons, with the following sizes: 50, 200, 400, 600, and 800 neurons. For each random sample, we applied FARCI and the other algorithms, and for each network size, we evaluated the average of AUROC and AUPR and the computational runtime.

**Figure 5:**
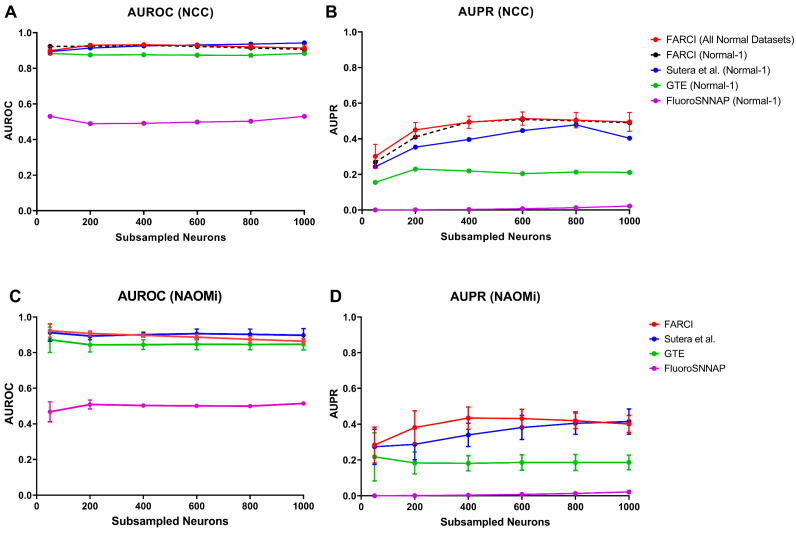
Performance evaluation of connectome inference with missing neurons. Comparison of FARCI with Sutera et al. GTE and FluoroSNNAP in terms of (**A**,**C**) AUROC and (**B**,**D**) AUPR using subsampled datasets from (**A**,**B**) NCC and (**C**,**D**) NAOMi datasets. Error bars indicate 95% confidence interval.

Figure 5A,B depicts the AUROC and AUPR from missing neurons simulations using the subsampled NCC datasets (generated using Normal-1 network). The results show that FARCI is able to maintain high AUROCs and AUPRs, even with up to 60% missing neurons in the dataset. While the AUROCs stay high (>0.9), the AUPRs drop quickly at >80% missing neurons. Both FARCI and Sutera et al. provided comparable AUROCs, but FARCI consistently gave higher AUPRs across different fractions of subsampling than Sutera et al. algorithm. GTE gave lower AUROCs and AUPRs than FARCI, but interestingly, its performance was stable across subsampling sizes. As expected, FluoroSNNAP provided the lowest scores among the algorithms across different subsampling sizes. Figure 5A,B also summarizes the performance of FARCI for each of the 1000-neuron networks in the NCC (*n* = 10), confirming the robustness of FARCI to missing neurons up to 60–80% of the connectome.

The results from random connectome subsampling of NAOMi datasets confirm the trends observed in the NCC datasets. As shown in Figure 5C,D, FARCI maintained robustly high AUROCs and AUPRs, up to 60% missing neurons. While Sutera et al. algorithm provided high AUROCs, comparable to FARCI, its AUPRs dropped with the percentages of missing neurons more quickly than FARCI. GTE algorithm again gave lower AUROCs and AUPRs than partial correlations (FARCI and Sutera et al.), but its performance was notably stable across different fractions of missing neurons. As before, FluoroSNNAP produced low AUROCs and AUPRs across all percentages of missing neurons.

### 3.7. Computational Speed

**Figure 6:**
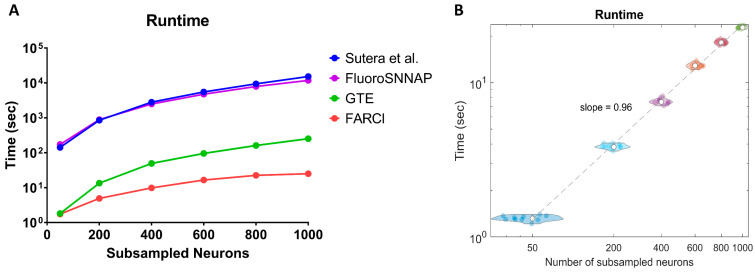
Computational runtimes for the NCC datasets. (**A**) Runtime comparison of FARCI, Sutera et al. GTE, and FluoroSNNAP algorithms using Normal-1 network. (**B**) FARCI runtimes for different sizes of subsampled networks for 1000-neuron datasets from the NCC (*n* = 10).

Besides accuracy, computational efficiency is a desirable feature of a connectome inference algorithm. The computational times in Figure 6A show that FARCI offered 2–3 orders of magnitude of computational speed-up over Sutera et al.’s algorithm over various network sizes. In addition, the computational runtimes of FARCI had a better scaling with network size than Sutera et al.’s algorithm—that is, a lower fold increase in computational times with increasing connectome size. GTE matched FARCI in computational speed for the smallest connectome (*n* = 50), but its computational scaling with connectome size was worse than FARCI. Finally, the computational cost and scaling of FluoroSNNAP approximately equaled that of Sutera et al.’s algorithm. The fast computational performance of FARCI is consistently observed across the datasets in the NCC, as shown in Figure 6B. Furthermore, Figure 6B indicates that the runtime of FARCI scales linearly with the size of the connectome, even to 10,000 neurons (see Supplementary Figure S3).

We noted that the most time-consuming step in FARCI was due to the spike deconvolution (i.e., OASIS), followed by the spike smoothing step (see Supplementary Figure S4). Both of these steps have a linear computational complexity scaling with the number of neurons—keeping the same number of frames—as they are applied to the activity data of each neuron separately. The calculation of partial correlation coefficients, however, has a cubic complexity scaling with respect to the number of neurons, which is associated with the inversion of the correlation matrix. We expect that beyond a certain population size, the calculation of partial correlations will become the most time-consuming.

## 4. Discussion

In this work, we developed FARCI, a fast and robust procedure for inferring functional neuronal connectome from two-photon Ca^2+^ imaging data. FARCI combines a fast non-negative deconvolution algorithm OASIS [22], spike thresholding, and spike smoothing, to extract information for neuronal spike events from Ca^2+^ fluorescence signals. FARCI produces a partial correlation network of the neurons for functional connectome inference. We benchmarked FARCI using in silico ground truth datasets from the Neural Connectomics Challenge [17] and by the state-of-the-art simulator NAOMi [18], and compared its performance with the winning algorithm in the NCC by Sutera et al. [13], Generalized Transfer Entropy [15], and Fluorescence Single Neuron and Network Analysis Package [19]. The results showed that FARCI outperforms the comparative methods in terms of connectome inference accuracy as measured by AUROC and AUPR and computational runtimes and scaling. FARCI and Sutera et al. methods provided AUROC values that are generally high (mostly above 0.8), and are better than GTE and FluoroSNNAP. Of note, FluoroSNNAP consistently gave the lowest scores that were similar to a random predictor (AUROC of 0.5). As the ground truth connectomes were sparse, we had imbalanced classes with many more negatives than positives. In this case, AUROC is known to be a poor diagnostic tool for method performance, and AUPR is the more sensitive metric for method performance. In terms of AUPR, FARCI outperformed; it had a slight advantage over the Sutera et al. algorithm and was superior to GTE and FluoroSNNAP. Also, the high performance of FARCI is robust with respect to the connectome size, data noise and sampling rate, and network densities.

Further, we demonstrated that FARCI performs well in the realistic scenario where there are missing neurons in the connectome inference. In this scenario, partial correlations between any two neurons may appear because they shared a hidden pre-synaptic neuron that is not part of the measurement. In our tests, FARCI was able to maintain high AUROC and AUPR up to 60% of missing neurons in a connectome of 1000 neurons, while still keeping moderately high AUPR until 80% of neurons missing. Sutera et al. algorithm also gave high AUROCs over different fractions of missing neurons, but its AUPR dropped more quickly than FARCI beyond 20% missing neurons. Among the comparative methods, GTE was notable. Despite having lower AUROCs and AUPRs than FARCI and Sutera et al. the performance of GTE was robust to missing neurons as the scores remained largely equal over the entire tested range (down to 95% missing neurons).

As noted above, FARCI produces a partial correlation network for the representation of the functional connectome. Partial correlations are symmetric and thus do not give an indication for the directionality of the neuronal connections—that is, no information regarding the identity of the pre- and post-synaptic neurons. Nevertheless, undirected functional connectomes inferred from neuronal activity recordings can facilitate understanding how functional connectomes are rewired during learning and memory formation. Besides, there are other limitations in determining directionality in neuronal connectome from two-photon Ca^2+^ fluorescence data. First, the typical rate of data sampling for two-photon Ca^2+^ imaging ranges between 30–100 ms (i.e., ~10–30 Hz) [35], which is much longer than the time scale of neuronal action potentials and the following refractory period between 1–5 ms [36]. Given the sampling rate of Ca^2+^ imaging, neurons may have fired several times in between any two image frames, and thus the expected sequential timing of pre- and post-synaptic neuron firing has a low chance to be captured accurately. Besides, because of the temporal coding scheme of neurons, the most informative data for establishing causal connections may reside in brief periods of time when the relevant neurons are active. While model-free methods for establishing causal connections using Ca^2+^ imaging data exist in the literature (e.g., using the concept of transfer entropy [15]), fundamental challenges in determining directional (causal) connectivity from time series data, like the ones mentioned above, will put a limit to the accuracy of the inferred connectome [6].

Besides, the use of simulated datasets for benchmarking may mask certain methodological limitations. First, the NCC datasets only included simulated activity of excitatory neurons. Any potential methodological bias related to inhibitory neurons and responses to their activity would therefore not appear in the scoring. In this regard, non-negativity assumption that is taken in OASIS method in FARCI for inferring Ca^2+^ spikes is known to lead to omission of neuronal response to inhibition [37]. Note that such an issue afflicts most of the current spike inference algorithms, but there are possible workarounds for connectomes in which inhibited neurons play a major role [37].

## 5. Conclusions

We presented FARCI, a MATLAB toolbox for reconstructing functional connectome using two-photon Ca^2+^ fluorescence data. FARCI relies on multivariate partial correlation analysis of (pre-processed) neuronal Ca^2+^ spike activity to establish connectivity among neurons. We benchmarked FARCI using in silico time-series Ca^2+^ fluorescence datasets from the Neural Connectomics Challenge [17] and those generated by the state-of-the-art simulator NAOMi [18], against the winning method in the NCC by Sutera et al. [13], Generalized Transfer Entropy [15], and FluoroSNNAP [19]. The results demonstrated the superior performance of FARCI, both in accuracy and computational time and scaling, over the comparative methods. However, FARCI produces a partial correlation network as its output, and thus does not provide the directionality of the neuronal connections. Also, like many existing inference methods, FARCI does not account for the activity of inhibitory neurons during the reconstruction of functional connectome.

## Figures and Tables

**Table 1:** Datasets provided in the Neural Connectomics Challenge. Each dataset contains three types of information: 1. neuronal activity in the form of Ca fluorescence signals, 2. the ground truth connectome structure, and 3. the spatial coordinates of neurons.

Networks	# of Neurons	Description
small (6 datasets)	100	six connectomes with 100 neurons
normal (4 datasets)	1000	four connectomes with 1000 neurons
normal3-highrate	1000	normal-3 connectome with a higher neuronal firing frequency
normal4-lownoise	1000	normal-4 connectome with a higher signal-to-noise ratio
highcc	1000	a connectome of 1000 neurons with a higher clustering coefficient
lowcc	1000	a connectome of 1000 neurons with a lower clustering coefficient
highcon	1000	a connectome of 1000 neurons with a higher average connectivity
lowcon	1000	a connectome of 1000 neurons with a lower average connectivity

**Table 2:** Effect of different signal processing steps on connectome inference. Unlike binarization, signal thresholding and smoothing improve the accuracy of connectome inference.

	AUROC			AUPR		
Filter	Small (*n* = 6)	Normal (*n* = 4)	Others (*n* = 6)	Small (*n* = 6)	Normal (*n* = 4)	Others (*n* = 6)
Neuronal Spikes x	0.871 ± 0.058	0.892 ± 0.002	0.905 ± 0.030	0.543 ± 0.097	0.335 ± 0.005	0.347 ± 0.067
Spikes + Binarization u(x)	0.563 ± 0.055	0.653 ± 0.006	0.647 ± 0.069	0.189 ± 0.021	0.042 ± 0.002	0.050 ± 0.021
Spikes + Thresholding g(x)	0.876 ± 0.051	0.882 ± 0.003	0.895 ± 0.040	0.620 ± 0.096	0.408 ± 0.012	0.421 ± 0.114
Spikes + Smoothing h(x)	0.891 ± 0.035	0.908 ± 0.002	0.917 ± 0.025	0.538 ± 0.056	0.330 ± 0.004	0.346 ± 0.053
FARCI h(g(x))	**0.916 ± 0.031**	**0.908 ± 0.002**	**0.918 ± 0.032**	**0.741 ± 0.067**	**0.491 ± 0.003**	**0.497 ± 0.100**

## Data Availability

The codes: user manual, and tutorial of FARCI are available online (https://github.com/CABSEL/FARCI and http://www.projectmemonet.org/farci, accessed on 19 November 2021).

## Supplementary Materials

The following are available online at https://www.mdpi.com/article/10.3390/brainsci11121556/s1, Figure S1: FARCI performance using spike thresholding based on percentiles (90th and 95th percentile). Figure S2: FARCI performance on downsampled datasets (25 Hz). Figure S3: FARCI average runtimes for connectomes larger than 1000-neuron datasets from the NCC (*n* = 10). Figure S4: FARCI runtimes for the NCC connectomes with 1000 neurons. Table S1: Parameters used to generate in-silico datasets using the NAOMi toolbox [1]. Table S2: Effect of different signal processing steps on connectome inference. Table S3: Effect of different signal processing steps on connectome inference. Table S4: Effect of different signal processing steps on connectome inference. Table S5: Alternative smoothing functions. Table S6: Performance comparison using different smoothing functions. Table S7: Comparison of FARCI performance with Sutera et al. GTE and FluoroSNNAP on all the datasets provided in Neural Connectomics Challenge. Table S8: Comparison of FARCI performance with Sutera et al. GTE and FluoroSNNAP on all the datasets generated using NAOMi Toolbox [1]. Table S9: Test of Normality and Equality of Variance.
