# Supplementary material for: FARCI: Fast and Robust Connectome Inference
Source: Brain Sci. 2021 Nov 24;11(12):1556. doi: 10.3390/brainsci11121556 (PMC8699247; doi:10.3390/brainsci11121556)
Supplement: Supplementary file 1 [file brainsci-11-01556-s001.zip › brainsci-1313151-supplementary.pdf]

Supplementary Materials

FARCI: Fast and Robust Connectome Inference

Saber Meamardoost[1], Mahasweta Bhattacharya[2], Eun Jung Hwang[3,4], Takaki Komiyama[3], Claudia Mewes[5], Linbing Wang[6], Ying Zhang[7], Rudiyanto Gunawan[1]

Table S1. Parameters used to generate *in-silico* datasets using the NAOMi toolbox [1].

Parameters	100 Neurons	1000 Neurons	Remarks
Dimensions of neural volume	50 µm × 50 µm × 150 µm	300 µm × 300 µm × 150µm	Dimensions are selected to generate approximately 100 and 1000 neurons
Imaging depth below surface	150 µm	150 µm	This is a typical depth in calcium imaging experiment
Number of frames	180K	180K	The total frames correspond to 50Hz for a total of 1-hour recording
Imaging rate	50Hz	50Hz	The same rate used in the NCC data generation
Calcium indicator	GCaMP6	GCaMP6	Default setting in NAOMi
Numerical aperture of objective	0.8	0.8	Default setting in NAOMi
Numerical aperture of excitation	0.6	0.6	Default setting in NAOMi
Power (mW)	40	40	Default setting in NAOMi
Motion consideration for scanning	'true'	'true'	Default setting in NAOMi

Table S2. Effect of different signal processing steps on connectome inference. AUROC and AUPR are reported for small datasets.

	AUROC						AUPR					
**Filter**	**small-1**	**small-2**	**small-3**	**small-4**	**small-5**	**small-6**	**small-1**	**small-2**	**small-3**	**small-4**	**small-5**	**small-6**
Spikes x	0.781	0.827	0.872	0.901	0.907	0.938	0.412	0.460	0.516	0.592	0.609	0.667
Spikes + Binarization $u(x)$	0.542	0.521	0.533	0.558	0.554	0.673	0.204	0.182	0.170	0.180	0.171	0.224
Spikes + Thresholding $g(x)$	0.795	0.839	0.879	0.903	0.910	0.933	0.480	0.545	0.604	0.671	0.689	0.733
Spikes + Smoothing $h(x)$	0.849	0.859	0.881	0.903	0.913	0.942	0.486	0.489	0.501	0.554	0.571	0.625
**FARCI** $\boldsymbol{h(g(x))}$	**0.876**	**0.890**	**0.910**	**0.928**	**0.934**	**0.960**	**0.667**	**0.686**	**0.700**	**0.767**	**0.787**	**0.838**

Table S3. Effect of different signal processing steps on connectome inference. AUROC and AUPR are reported for normal datasets.

	AUROC				AUPR			
**Filter**	**normal-1**	**normal-2**	**normal-3**	**normal-4**	**normal-1**	**normal-2**	**normal-3**	**normal-4**
Spikes x	0.891	0.894	0.892	0.889	0.334	0.337	0.342	0.329
Spikes + Binarization $u(x)$	0.656	0.655	0.655	0.643	0.042	0.043	0.044	0.040
Spikes + Thresholding $g(x)$	0.878	0.885	0.884	0.880	0.395	0.413	0.422	0.403
Spikes + Smoothing $\mathrm{h}(x)$	0.910	0.909	0.910	0.906	0.336	0.327	0.332	0.327
**FARCI** $\boldsymbol{h(g(x))}$	**0.907**	**0.909**	**0.910**	**0.905**	**0.490**	**0.491**	**0.495**	**0.488**

Table S4. Effect of different signal processing steps on connectome inference.

	AUROC						AUPR					
**Filter**	**normal-3 (highrate)**	**normal-4 (lownoise)**	**highcc**	**lowcc**	**highcon**	**lowcon**	**normal-3 (highrate)**	**normal-4 (lownoise)**	**highcc**	**lowcc**	**highcon**	**lowcon**
Spikes x	0.937	0.919	0.907	0.870	0.867	0.930	0.409	0.366	0.416	0.233	0.321	0.334
Spikes + Binarization $u(x)$	0.742	0.625	0.707	0.569	0.579	0.660	0.091	0.039	0.054	0.028	0.046	0.044
Spikes + Thresholding $g(x)$	0.935	0.914	0.900	0.859	0.835	0.927	0.533	0.507	0.509	0.271	0.298	0.407
Spikes + Smoothing $h(x)$	0.945	0.913	0.912	0.888	0.894	0.949	0.405	0.337	0.392	0.255	0.334	0.350
**FARCI** $\boldsymbol{h}(\boldsymbol{g}(\boldsymbol{x}))$	**0.953**	**0.915**	**0.916**	**0.892**	**0.875**	**0.956**	**0.602**	**0.532**	**0.575**	**0.380**	**0.368**	**0.525**

Table S5. Alternative smoothing functions. Smoothing functions F_1 to F_4 are obtained from [2].

Gaussian filter: $f(y_i, t) = \frac{1}{\sqrt{2\pi}\sigma} e^{-\frac{{y_i^t}^2}{2\sigma^2}},\ \sigma = 0.5$
$f_1(y_i, t) = y_i^{t-1} + y_i^t + y_i^{t+1}$
$f_2(y_i, t) = 0.4y_i^{t-3} + 0.6y_i^{t-2} + 0.8y_i^{t-1} + y_i$
$f_3(y_i, t) = y_i^{t-1} + y_i^t + y_i^{t+1} + y_i^{t+2}$
$f_4(y_i, t) = y_i^t + y_i^{t+1} + y_i^{t+2} + y_i^{t+3}$

Table S6. Performance comparison using different smoothing functions. The smoothing functions are provided in Supplementary Table S5. The window size for moving average is 5 frames.

	FARCI		Gaussian filter		Moving Average		F1 filter		F2 filter		F3 filter		F4 filter		Sutera *et al.* (2015)	
	**AUROC**	**AUPR**	**AUROC**	**AUPR**	**AUROC**	**AUPR**	**AUROC**	**AUPR**	**AUROC**	**AUPR**	**AUROC**	**AUPR**	**AUROC**	**AUPR**	**AUROC**	**AUPR**
small1	0.876	0.667	0.784	0.418	0.867	0.644	0.864	0.625	0.865	0.629	0.867	0.638	0.867	0.638	0.720	0.401
small2	0.890	0.686	0.827	0.465	0.883	0.662	0.884	0.660	0.884	0.659	0.885	0.663	0.885	0.663	0.791	0.490
small3	0.910	0.700	0.859	0.496	0.905	0.684	0.909	0.686	0.908	0.685	0.908	0.687	0.908	0.687	0.859	0.598
small4	0.928	0.767	0.878	0.523	0.926	0.757	0.928	0.755	0.927	0.756	0.927	0.758	0.927	0.758	0.895	0.717
small5	0.934	0.787	0.892	0.544	0.933	0.778	0.933	0.770	0.933	0.773	0.933	0.777	0.933	0.777	0.919	0.751
small6	0.960	0.838	0.914	0.572	0.960	0.831	0.958	0.816	0.959	0.821	0.960	0.827	0.960	0.827	0.960	0.845
normal1	0.907	0.490	0.888	0.319	0.905	0.482	0.912	0.487	0.912	0.487	0.910	0.487	0.910	0.487	0.943	0.403
normal2	0.909	0.491	0.892	0.324	0.909	0.483	0.916	0.495	0.916	0.495	0.914	0.492	0.914	0.492	0.942	0.404
normal3	0.910	0.495	0.892	0.329	0.908	0.488	0.916	0.498	0.915	0.498	0.913	0.496	0.913	0.496	0.942	0.398
normal4	0.905	0.488	0.890	0.320	0.904	0.480	0.912	0.488	0.911	0.488	0.909	0.487	0.909	0.487	0.939	0.388
normal3-highrate	0.953	0.602	0.940	0.422	0.949	0.590	0.958	0.607	0.957	0.604	0.954	0.601	0.954	0.601	0.961	0.452
normal4-lownoise	0.915	0.532	0.917	0.383	0.912	0.523	0.926	0.550	0.923	0.544	0.919	0.538	0.919	0.538	0.941	0.432
highcc	0.916	0.575	0.905	0.390	0.915	0.568	0.923	0.576	0.922	0.575	0.919	0.574	0.919	0.574	0.946	0.486
lowcc	0.892	0.380	0.872	0.243	0.890	0.367	0.900	0.380	0.899	0.379	0.896	0.377	0.896	0.377	0.925	0.260
highcon	0.875	0.368	0.846	0.283	0.874	0.364	0.880	0.383	0.881	0.382	0.879	0.378	0.879	0.378	0.944	0.413
Lowcon	0.956	0.525	0.937	0.296	0.954	0.512	0.961	0.520	0.960	0.520	0.959	0.519	0.959	0.519	0.955	0.334

Table S7. Comparison of FARCI performance with Sutera *et al.* (2015), GTE and FluoroSNNAP on all the datasets provided in Neural Connectomics Challenge.

	**FARCI**		**Sutera *et al.* (2015)**		**Generalized Transfer Entropy**		**FluoroSNNAP**	
	**AUROC**	**AUPR**	**AUROC**	**AUPR**	**AUROC**	**AUPR**	**AUROC**	**AUPR**
small1	0.876	0.667	0.720	0.401	0.648	0.308	0.534	0.201
small2	0.890	0.686	0.791	0.490	0.709	0.379	0.518	0.181
small3	0.910	0.700	0.859	0.598	0.787	0.458	0.527	0.165
small4	0.928	0.767	0.895	0.717	0.824	0.559	0.526	0.165
small5	0.934	0.787	0.919	0.751	0.880	0.605	0.526	0.157
small6	0.960	0.838	0.960	0.845	0.919	0.623	0.534	0.154
normal1	0.907	0.490	0.943	0.403	0.874	0.230	0.527	0.022
normal2	0.909	0.491	0.942	0.404	0.879	0.241	0.530	0.022
normal3	0.910	0.495	0.942	0.398	0.876	0.258	0.528	0.022
normal4	0.905	0.488	0.939	0.388	0.856	0.200	0.530	0.022
normal3-highrate	0.953	0.602	0.961	0.452	0.888	0.266	0.532	0.022
normal4-lownoise	0.915	0.532	0.941	0.432	0.863	0.209	0.529	0.022
highcc	0.916	0.575	0.946	0.486	0.878	0.234	0.534	0.022
lowcc	0.892	0.380	0.925	0.260	0.840	0.137	0.516	0.023
highcon	0.875	0.368	0.944	0.413	0.898	0.290	0.515	0.037
lowcon	0.956	0.525	0.955	0.334	0.700	0.113	0.544	0.015

Table S8. Comparison of FARCI performance with Sutera *et al.* (2015), GTE and FluoroSNNAP on all the datasets generated using NAOMi Toolbox [1].

		FARCI		Sutera *et al.* (2015)		Generalized Transfer Entropy		FluoroSNNAP	
	**Neurons**	**AUROC**	**AUPR**	**AUROC**	**AUPR**	**AUROC**	**AUPR**	**AUROC**	**AUPR**
naomi-1	106	0.854	0.622	0.776	0.450	0.706	0.368	0.508	0.178
naomi-2	118	0.827	0.555	0.806	0.469	0.625	0.226	0.520	0.162
naomi-3	102	0.860	0.638	0.721	0.407	0.651	0.309	0.534	0.202
naomi-4	113	0.829	0.545	0.788	0.429	0.719	0.331	0.500	0.155
naomi-5	104	0.854	0.602	0.858	0.556	0.651	0.230	0.514	0.158
naomi-6	1016	0.870	0.426	0.918	0.458	0.871	0.220	0.514	0.021
naomi-7	1023	0.850	0.432	0.885	0.463	0.861	0.209	0.510	0.020
naomi-8	1009	0.850	0.372	0.850	0.333	0.805	0.171	0.515	0.021
naomi-9	1027	0.870	0.418	0.910	0.439	0.855	0.192	0.514	0.021
naomi-10	1013	0.880	0.352	0.923	0.377	0.839	0.137	0.509	0.023

Table S9. Test of Normality and Equality of Variance. The tests were done to check normality and equality of variance assumptions for the differences in AUROCs and AUPRs for FARCI vs. Sutera *et al.*, FARCI vs. GTE, and FARCI vs. FluoroSNNAP. The normality test was done using a one-sample Kolmogorov-Smirnov (KS) test using the `kstest` function in MATLAB [3]. The constant variance test was done in 10 replicates using two-sample F-test applied to two random halves of the sample using the `vartest2` in MATLAB [4], to obtain an average *p*-value. The results suggest that constant variance assumption generally cannot be rejected at *p*-value<0.01. In some cases, among the NCC datasets, normality test can be rejected, particularly for FARCI vs. FluoroSNNAP.

		**NCC**				**NAOMi**			
		**AUROC**		**AUPR**		**AUROC**		**AUPR**	
		100 Neurons	1000 Neurons	100 Neurons	1000 Neurons	100 Neurons	1000 Neurons	100 Neurons	1000 Neurons
Kolmogorov-Smirnov Test	FARCI vs Sutera *et al.*	0.066	0.008	0.068	0.012	0.114	0.112	0.090	0.130
	FARCI vs GTE	0.051	0.010	0.022	0.004	0.064	0.117	0.037	0.040
	FARCI vs FluoroSNNAP	0.007	0.000	0.003	0.000	0.022	0.117	0.019	0.019
F-test	FARCI vs Sutera *et al.*	0.501	0.179	0.419	0.236	0.469	0.251	0.730	0.163
	FARCI vs GTE	0.649	0.012	0.352	0.229	0.246	0.523	0.478	0.454
	FARCI vs FluoroSNNAP	0.653	0.658	0.697	0.314	0.584	0.523	0.431	0.501

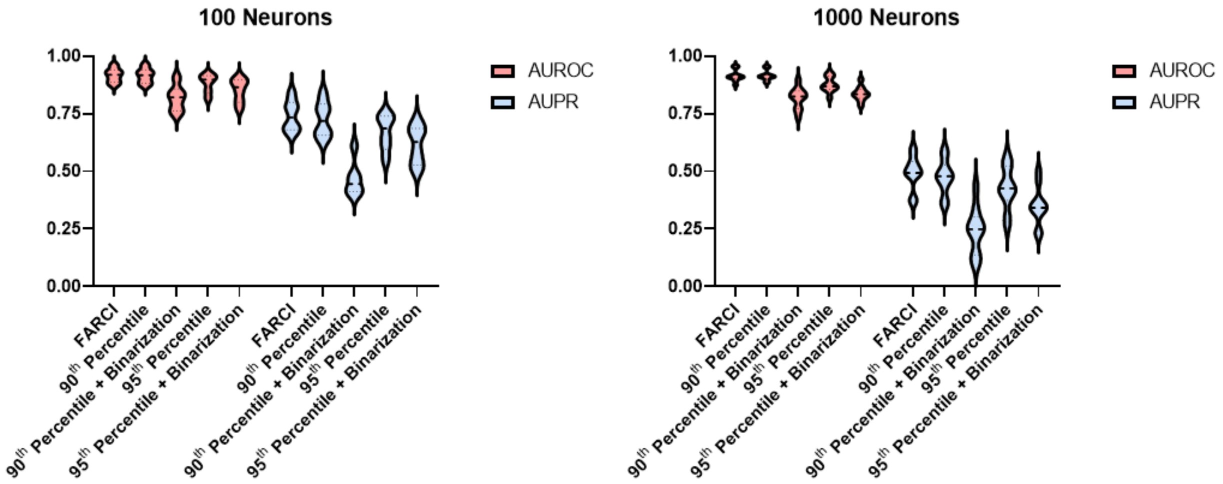

Figure S1. FARCI performance using spike thresholding based on percentiles (90th and 95th percentile). The performance of FARCI was assessed using AUROC and AUPR (see Materials and Methods).

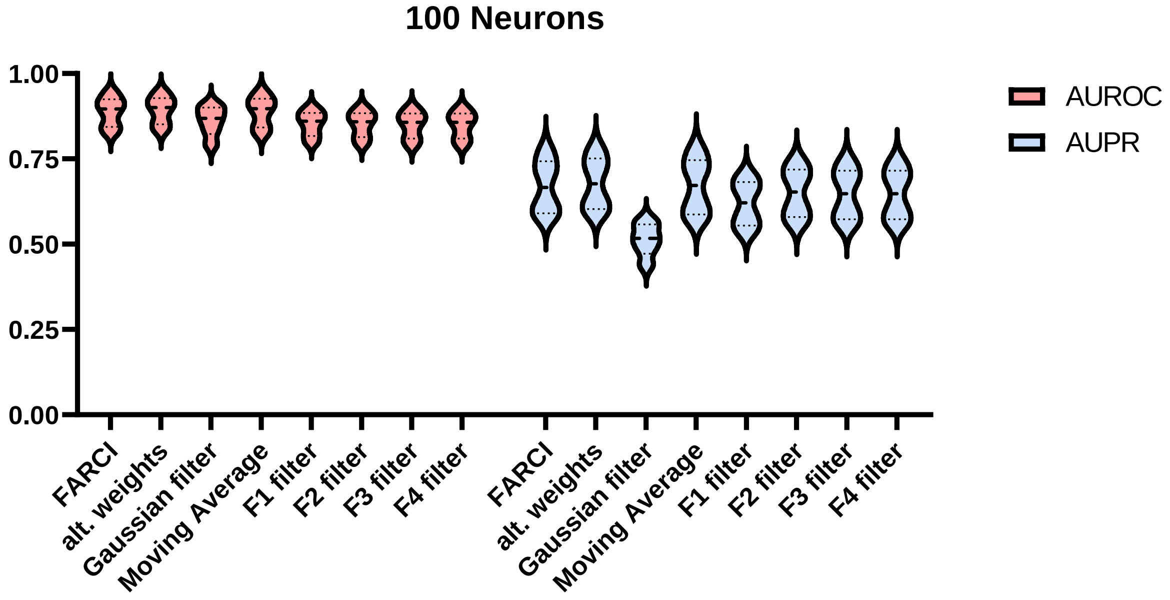

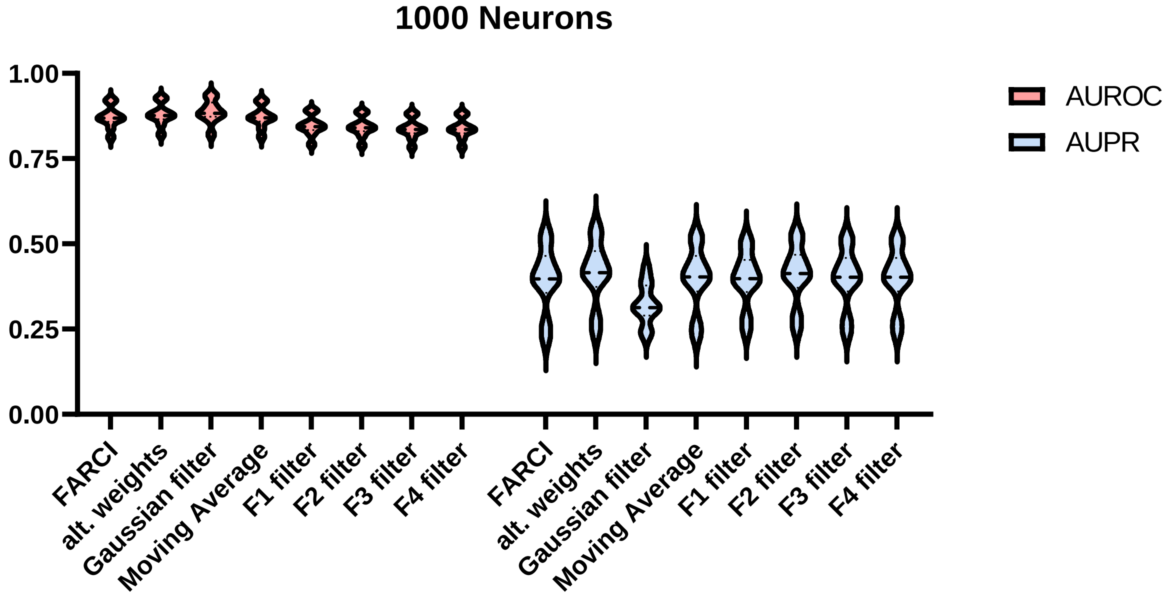

Figure S2. FARCI performance on downsampled datasets (25Hz). Alternative weights (alt. weights) refer to a spike smoothing function as follows: $h(y_i, t) = \frac{1}{5} y_i^{t-2} + \frac{3}{5} y_i^{t-1} + y_i^t + \frac{3}{5} y_i^{t+1} + \frac{1}{5} y_i^{t+2}$. The window size for moving average is 5. AUROCs and AUPRs for downsampled datasets are expectedly lower than for the original datasets due to the loss of information caused by downsampling. In general, the AUROCs and AUPRs remain relatively high.

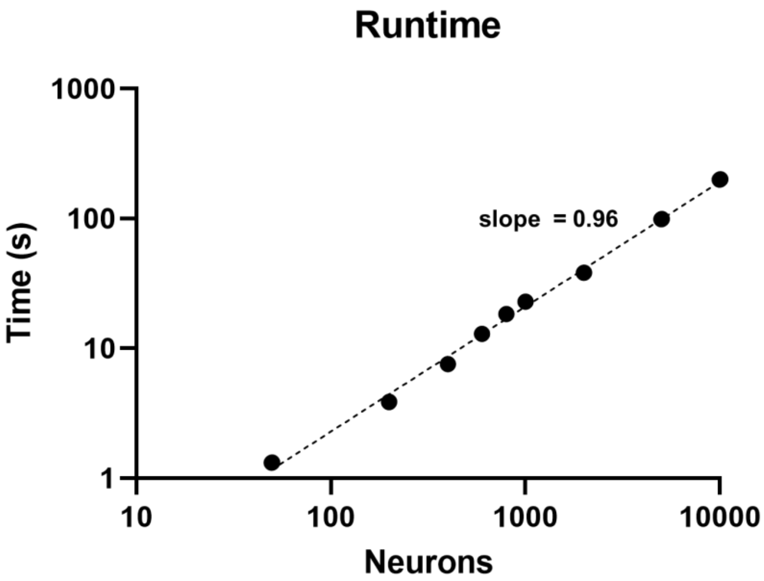

Figure S3. FARCI average runtimes for connectomes larger than 1000-neuron datasets from the NCC (n = 10). The average runtimes for subsampled networks <1000 neurons were obtained as described in the main text. For connectome larger than 1000 neurons, we combined mutiple normal datasets for 1000 neurons to produce datasets of size 2000, 5000, and 10000 neurons. As shown, the computational times scales linearly with the number of neurons.

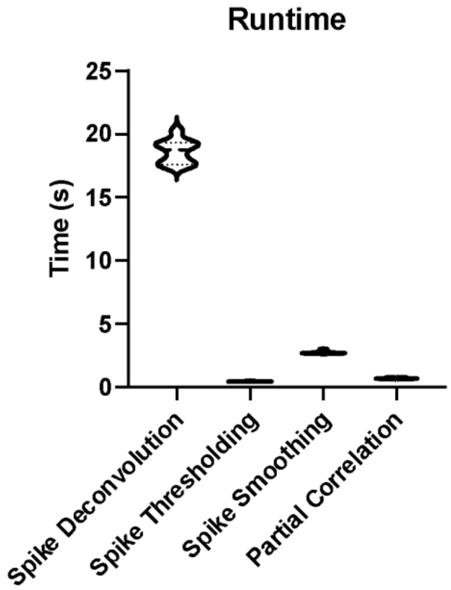

Figure S4. FARCI runtimes for the NCC connectomes with 1000 neurons.

References

1. Song, A.; Gauthier, J.L.; Pillow, J.W.; Tank, D.W.; Charles, A.S. Neural Anatomy and Optical Microscopy (NAOMi) Simulation for Evaluating Calcium Imaging Methods. *Journal of Neuroscience Methods* 2021, *358*, 109173, doi:10.1016/j.jneumeth.2021.109173.
2. Sutera, A.; Joly, A.; François-Lavet, V.; Qiu, Z.A.; Louppe, G.; Ernst, D.; Geurts, P.; Battaglia, D.; Guyon, I.; Lemaire, V.; et al. *Simple Connectome Inference from Partial Correlation Statistics in Calcium Imaging*; 2015; Vol. 46;.
3. One-Sample Kolmogorov-Smirnov Test - MATLAB Kstest - MathWorks Switzerland Available online: https://ch.mathworks.com/help/stats/kstest.html (accessed on 9 September 2021).
4. Two-Sample F-Test for Equal Variances - MATLAB Vartest2 - MathWorks Switzerland Available online: https://ch.mathworks.com/help/stats/vartest2.html (accessed on 9 September 2021).
